# Fabrication Processes to Generate Concentration Gradients in Polymer Solar Cell Active Layers

**DOI:** 10.3390/ma10050518

**Published:** 2017-05-09

**Authors:** Shusei Inaba, Varun Vohra

**Affiliations:** Department of Engineering Science, University of Electro-Communications, Chofu 182-8585, Japan; sys.1101.n1@gmail.com

**Keywords:** organic solar cells, conjugated polymer, bulk heterojunction, P3HT, PCBM, PCDTBT, PTB7

## Abstract

Polymer solar cells (PSCs) are considered as one of the most promising low-cost alternatives for renewable energy production with devices now reaching power conversion efficiencies (PCEs) above the milestone value of 10%. These enhanced performances were achieved by developing new electron-donor (ED) and electron-acceptor (EA) materials as well as finding the adequate morphologies in either bulk heterojunction or sequentially deposited active layers. In particular, producing adequate vertical concentration gradients with higher concentrations of ED and EA close to the anode and cathode, respectively, results in an improved charge collection and consequently higher photovoltaic parameters such as the fill factor. In this review, we relate processes to generate active layers with ED–EA vertical concentration gradients. After summarizing the formation of such concentration gradients in single layer active layers through processes such as annealing or additives, we will verify that sequential deposition of multilayered active layers can be an efficient approach to remarkably increase the fill factor and PCE of PSCs. In fact, applying this challenging approach to fabricate inverted architecture PSCs has the potential to generate low-cost, high efficiency and stable devices, which may revolutionize worldwide energy demand and/or help develop next generation devices such as semi-transparent photovoltaic windows.

## 1. Introduction

**Figure 1:**
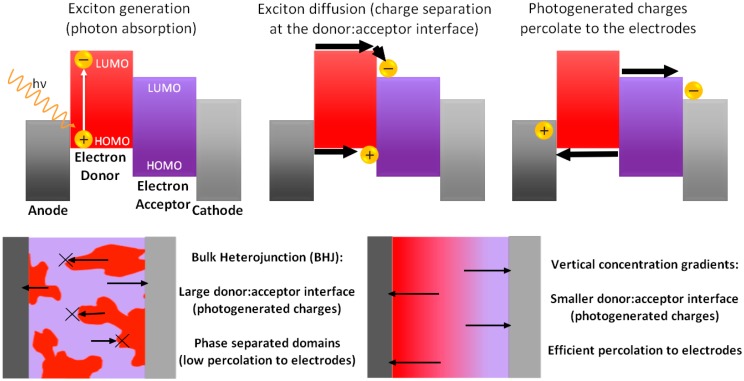
Working principle of charge separation at the electron donor-electron acceptor interface and charge transport to the respective electrodes depicted using phase separated or concentration graded active layers.

Since the pioneering work of Tang in 1986 [1], organic solar cells have been steadily improving their performances. In fact, both solution-processed small molecules and polymer solar cells (PSCs) now reach power conversion efficiencies (PCEs) over the milestone value of 10% [2,3,4,5,6,7,8,9]. Although these performances do not allow them to tackle the state-of-the-art silicon technologies yet, due to their low fabrication cost, lightweight and potential to be integrated into a variety of next-generation technologies such as wearable electronics or semi-transparent photovoltaic windows, PSCs have attracted great interest from the materials science community over the past decade [10,11,12,13,14]. The introduction and development of active layers composed of poly(3-hexylthiophene) (P3HT) and fullerene derivatives in the early 2000s is one of the first major achievements in the field as they led to a large increase in PCE over 6% [15,16]. Even a decade later, P3HT-based active layers were still considered as benchmark materials for the study and improvement of PSC performances [17]. However, P3HT-based active layers have now reached their limit and researchers have been focusing on developing new materials that allow for better light-harvesting and/or higher charge transport properties [18,19]. While developing new materials seems to be an efficient strategy to tune the photovoltaic parameters of PSCs, a large number of studies emphasize that a particular attention should be given to the active layer morphologies in order to fabricate high PCE devices [13,20]. For instance, the formation of electron donor (ED)- and acceptor (EA)-rich phase separated domains can be either extremely positive or negative depending on the size of the domains and their relative position with respect to the electrodes. This can be easily understood when taking into account the working principle of PSCs (Figure 1).

Once photons are absorbed in the active layer, the excitons with limited diffusion lengths on the nanometer scale reach an ED–EA interface to undergo charge separation leading to the formation of electrons and holes. Consequently, efficient exciton to photogenerated charges dissociation only occurs in the presence of a large ED–EA interface. However, once charges are generated, electron and holes will percolate to the cathode and the anode, respectively. This can be efficiently achieved in active layers possessing the adequate vertical ED–EA distribution resulting in devices with large short-circuit current densities (Jsc) and fill factors (FF). The open-circuit voltage (Voc) also benefits from an adequate vertical concentration gradient as Voc increases with decreasing reverse saturation current (J_0_) [21]. Note that these positive effects should be observed in PSCs with both regular (top cathode/bottom transparent anode) and inverted (top anode/bottom transparent cathode) device architectures. Nevertheless and independently of the achieved PCEs, inverted PSCs (iPSCs) should lead to higher device durability as, unlike regular PSCs (rPSCs), their top gold or silver electrodes are not easily oxidized [22].

Both theoretical and experimental results have demonstrated that ED–EA vertical concentration gradients will play a major role in the production of high efficiency devices, especially when a layered structure is obtained composed of an ED-rich layer on the anode side, an EA-rich layer on the cathode side and an intermixed layer sandwiched between the two first layers [23,24]. However, it is not always easy to fabricate such active layers, especially in inverted device architectures. Here, we will review the fabrication processes to generate such vertical concentration gradients in both single layer and sequentially deposited multilayer active layers. In particular, we will demonstrate that in single active layers, the interactions of the active materials with each other and with the interfacial layers can generate a variety of vertical concentration gradients which can then be tuned using thermal annealing. Additionally, as solubility of the ED and EA materials with respect to the used solvents may differ significantly, using drying kinetics and surface treatments or adjusting the chemical nature of the active materials are alternative valid strategies to generate the adequate vertical concentration gradients in both single layer rPSCs or iPSCs. However, depositing multilayer active layers in which each layer contains increasing concentrations of ED or EA molecules probably represents the most straightforward method for concentration gradients production. Although very simple in principle, in practice, this approach can become rather challenging, as top layers should be deposited without dissolving or damaging the underlying layers. Here, we will show some examples of studies in which multilayer fabrication was successfully achieved in iPSCs and rPSCs. While dry-transfer methods seem to be the most efficient approach for iPSCs, the proper selection of orthogonal solvents for sequential deposition in rPSCs may be possible. Furthermore, playing on the relative solubilities of polymer EDs and fullerene EAs, an innovative approach was developed to readily generate adequate concentration gradients through the formation of diffusive bilayer PSCs (DfBL-PSCs). After discussing the validity of this approach, we will examine whether this fabrication process has the potential to overcome the state-of-the-art active layer morphology referred to as bulk heterojunction (BHJ) which is obtained by co-depositing ED and EA molecules from the same solution. This approach, which has been, up to now, almost exclusively limited to rPSCs fabrication, seems to display improved PCEs and durability with respect to BHJ-PSCs.

## 2. Generation of Vertical ED–EA Distribution in Single Active Layer PSCs

### 2.1. Effect of Thermal Annealing and Interfacial Interactions

PSCs are among the most studied devices in organic electronics and numerous publications can be found introducing new materials which display enhanced opto-electrical properties. P3HT:fullerene derivative is undoubtedly a reference ED–EA pair, in particular when P3HT is associated with [6,6]-Phenyl-C_61_-Butyric Acid Methyl Ester (PC_61_BM) [17]. One particular aspect that has been extensively studied is the effect of processing parameters on the resulting morphology in P3HT:PC_61_BM thin films and active layers for PSCs [25]. While phase separation between the ED and EA materials was initially only considered in the horizontal direction of the active layer (in the plane parallel to the electrodes), with the development of new analytical techniques, probing the vertical ED–EA distribution has now become a common characterization in the field. These analytical techniques have been reviewed elsewhere and therefore, will not be presented here [14]. Various studies have confirmed that, in the pristine (as spun) state of BHJ active layers, a PC_61_BM-depleted layer is formed at the surface of the films; independently of the spin-coating speed [20,26,27,28]. While the deposition speed may not be of major importance, the substrate on which the P3HT:PC_61_BM layer is deposited highly influences the vertical ED–EA distribution in the film. The formation of P3HT-rich and PC_61_BM-rich layers, respectively, at the top and bottom of the thin film suggest that these active layers would be more suitable for iPSCs compared to rPSCs. In fact, after characterizing the thin films using X-ray photoelectron spectroscopy (XPS), Xu et al. demonstrated that for active layer deposited under the same conditions, regular architectures only exhibit PCEs of 0.74% while their inverted equivalents produced efficiencies up to 1.93% [26]. However, the same study also emphasizes the fact that these vertical profiles and the formation of depleted layers are highly dependent on whether or not the films are thermally annealed and on the chemical nature of the coated substrate.

**Figure 2:**
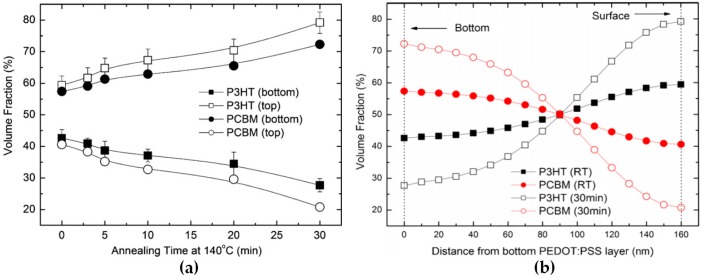
(**a**) Effect of annealing time at 140 °C on the poly(3-hexylthiophene):[6,6]-Phenyl-C_61_-Butyric Acid Methyl Ester (P3HT:PC_61_BM) compositions at the interfaces with poly(3,4-ethylenedioxythiophene) polystyrene sulfonate (PEDOT:PSS, bottom) and air (top); (**b**) Variation of vertical electron donor-electron acceptor distribution upon annealing at 140 °C for 30 min. Adapted from [27], with permission from © 2011 Elsevier.

Thermal annealing is a post-deposition process which remarkably improves the performances of P3HT:PC_61_BM based PSCs [20,21,29,30,31]. The improved performances are commonly ascribed to the diffusion of PC_61_BM along with the crystallization of the active materials leading to a sufficient phase separation and consequent formation of crystalline P3HT-rich and PC_61_BM-rich domains with enhanced charge transport properties [32,33]. The typical annealing temperature for this ED–EA system in rPSCs is 140 °C and the formation of depletion layers observed by spectroscopic ellipsometry (SE) upon annealing can be clearly correlated with the annealing time prior to electrode deposition when films are deposited on poly(3,4-ethylenedioxydithiophene):poly(styrene sulfonate), also known as PEDOT:PSS (Figure 2) [27]. Van Bavel et al. studied the effect of annealing at 130 °C for 20 min in rPSC devices in which the active layers with various thicknesses were annealed after deposition of the LiF/Al cathode [30]. Their results, obtained using electron tomography and summarized in Table 1, clearly emphasize that the active layer thickness and the annealing temperature influence the formation of adequate vertical concentration gradients for rPSCs. In devices with 100 nm-thick annealed active layers, an adequate P3HT vertical gradient is formed leading to FF as high as 62%, which, together with a Jsc and Voc of 9.4 mA/cm^2^ and 0.6 V, respectively, result in a PCE of 3.5%. These positive results for rPSCs, which disagree with most previously introduced works, may be related to the post-annealing cooling conditions and well correlate with devices prepared with slow-cooling conditions which exhibit similar PC_61_BM-depleted regions at the bottom of the active layer in rPSCs [34]. Another important parameter to be taken into account in van Bavel’s work is the top interface used during annealing (LiF/Al).

**Figure 3:**
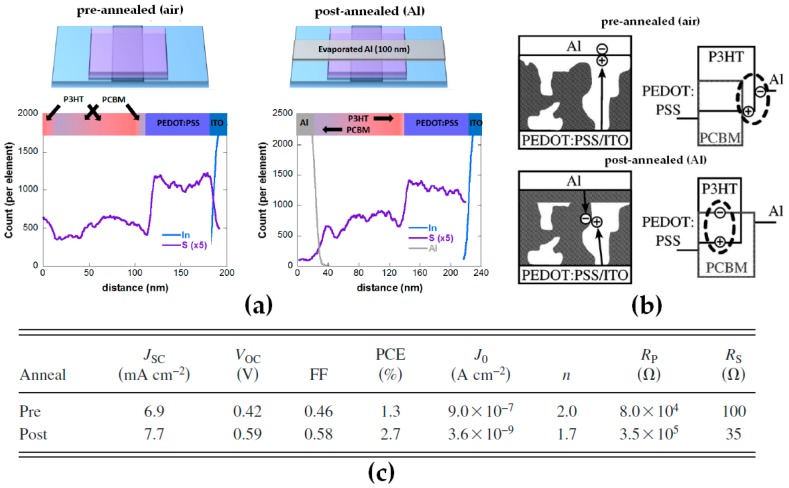
(**a**) Vertical concentration profiles and (**b**) schematic representation of bulk heterojunction active layers annealed in air and after deposition of the Al electrodes together with (**c**) their photovoltaic performances. Adapted from [20,21], with permission from © 2017 One Central Press and © 2010 American Institute of Physics.

As mentioned previously, the formation of ED or EA-depleted layers at the buried interface with the substrate is highly dependent on the chemical nature of the substrate [26,35]. Although a simple operation (spin-coating of a blend solution), active layer formation is a complicated process in which ED and EA molecules not only interact with each other but also with the solvent, the substrate on which they are deposited and the surrounding air. Similarly, during annealing, the top interface also plays an essential role as, depending on its interactions with the ED and EA molecules, it may induce positive or detrimental vertical distributions through the formation of ED or EA-depleted top layers [20,21,28,31]. In fact, several studies have demonstrated similar effects when it comes to annealing P3HT:PC_61_BM active layers in regular device architectures with a top aluminum cathode. The energy-dispersive X-ray spectroscopy (EDS) sulfur profiles in Figure 3a obtained by annealing the active without (pre-annealed) and with (post-annealed) a top aluminum cathode confirm that while annealing in air results in a PCBM-depleted layer at the interface between the active layer and air, this depleted layer evolves into a PCBM-rich layer when the thermal annealing is applied after deposition of the metal electrode [20]. The formation of a more adequate vertical concentration gradient consequently enhances the electron extraction properties at the active layer/cathode interface (Figure 3b) which improves all the photovoltaic parameters in rPSCs and a PCE of 2.7% is obtained for post-annealed devices as compared to 1.3% for pre-annealed devices (Figure 3c) [21].

**Figure 4:**
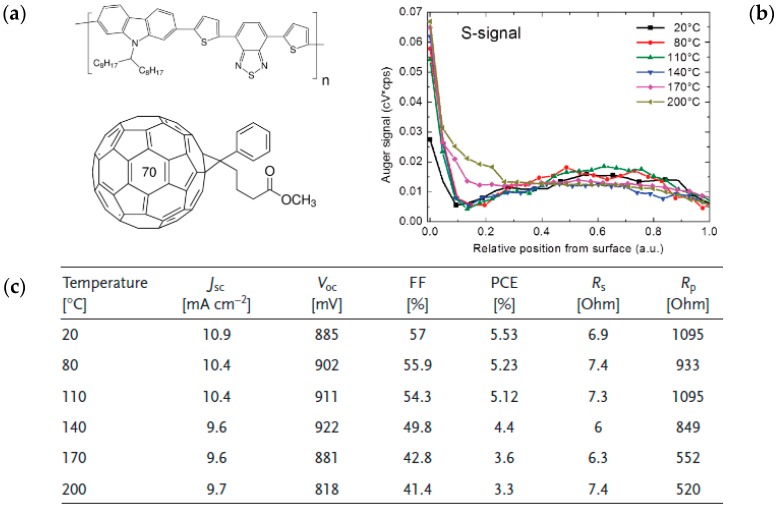
(**a**) Molecular structures of Poly[*N*-9′-heptadecanyl-2,7-carbazole-alt-5,5-(4′,7′-di-2-thienyl-2′,1′,3′-benzothiadiazole)] (PCDTBT) and [6,6]-Phenyl-C_71_-Butyric Acid Methyl Ester (PC_71_BM); (**b**) sulfur profiles and (**c**) device parameters of PCDTBT:PC_71_BM active layers deposited on poly(3,4-ethylenedioxythiophene) polystyrene sulfonate (PEDOT:PSS) and annealed in air at various temperatures. Adapted from [38], with permission from © 2013 WILEY-VCH.

Note that the effect of annealing and interfacial materials on the active layer morphology and vertical distribution is not limited to the P3HT:PC_61_BM pair but has also been studied in active layers based on highly efficient solution-processed EDs [36,37,38,39,40]. Poly[*N*-9′-heptadecanyl-2,7-carbazole-alt-5,5-(4′,7′-di-2-thienyl-2′,1′,3′-benzothiadiazole)] (PCDTBT) and its derivatives have been increasingly studied since 2007 and devices based on PCDTBT paired with fullerene derivatives now often exhibit PCEs over 6% [41,42,43,44]. However, when it comes to the unsubstituted PCDTBT:[6,6]-Phenyl-C_71_-Butyric Acid Methyl Ester (PC_71_BM), similar to P3HT:PC_61_BM active layers, Auger electron spectroscopy measurements reveal that EA-depleted layers can be found at the surface of the thin films which are detrimental for rPSC device architectures (Figure 4) [38]. Thermal annealing of the PCDTBT:PC_71_BM active layers at temperatures up to 200 °C does not have a positive effect on the resulting ED–EA concentration gradient and, consequently, to generate the adequate vertical concentration gradients for rPSCs, a new type of annealing, namely solvent annealing, was introduced.

### 2.2. Solvent Assisted Vertical Molecular Distribution

**Figure 5:**
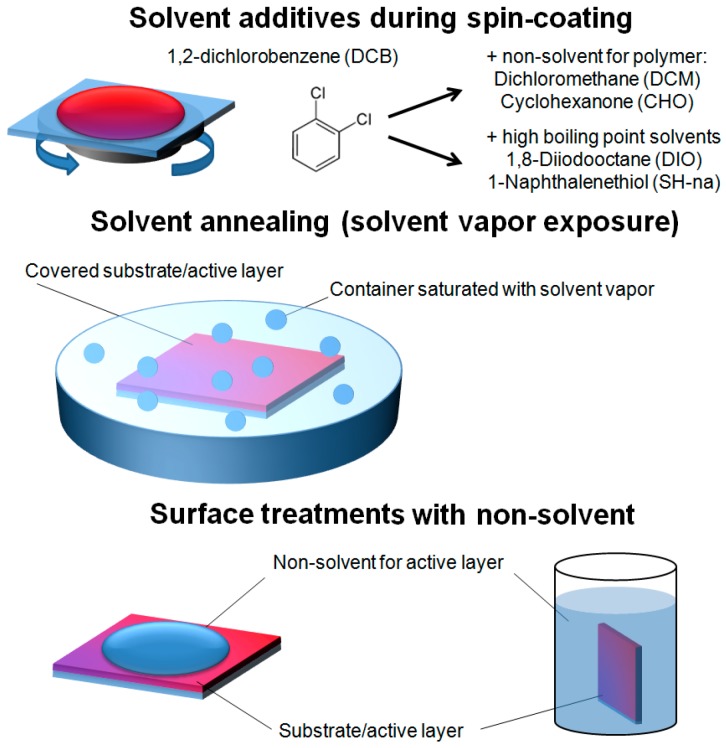
Schematic representation of the solvent processes to induce vertical concentration gradients in PSC active layers.

Solvent annealing is an alternative post-deposition method to improve the morphologies of active layers for BHJ-PSCs [45]. The results presented by Wang et al. clearly emphasize that a short time (60 s) solvent annealing of poly[(4,8-bis-(2-ethylhexyloxy)-benzo[1,2-b:4,5-b0](dithiophene)-2,6-diyl-alt-(4-(2-ethylhexanoyl)-thieno[3,4-b]thiophene)-2,6-diyl]:PC71BM using chloroform vapors can increase the photovoltaic performances of rPSCs exhibiting enhanced Jsc and FF along with higher shunt resistances (Rsh) and lower series resistances (Rs). The evolution of these parameters suggests that adequate vertical ED–EA distribution for regular architecture was achieved through solvent annealing of the active layers. Furthermore, in this study, the authors emphasize that the use of mixed solvents or high boiling point solvent additives such as 1,8-diiodoctane (DIO) can further increase the device performances and, using a combination of the three methods, they were able to fabricate regular architecture devices with PCEs up to 7.58%. Solvent-based processes are commonly associated with the difference in solubility of the polymer EDs and fullerene EAs in the solvents used for active layer deposition and post-deposition treatments such as solvent annealing or surface treatment with non solvents [46,47,48,49,50,51,52,53,54]. Here, we will compare the results from the various solvent-processes inducing changes in vertical distribution. These can be separated into three categories, namely, use of mixed solvents with different boiling temperatures (or solvent additives), solvent annealing and surface treatment, which are schematized in Figure 5. The use of mixed solvents can either take advantage of the difference in solubility of the ED and EA materials in the two solvents or more simply modify the drying kinetics of the system by using higher or lower boiling point solvents. Using non-solvents for one of the materials (e.g., the polymer ED) also corresponds to an interesting strategy to induce preferential crystallization of the polymer resulting in a polymer-depleted top interface in the active layers. This was performed in two studies using dichloromethane (DCM) or cyclohexanone (CHO) as solvent additive in 1,2-dichlorobenzene (DCB)-based P3HT:PC_61_BM solutions [47,48]. DCM is a low boiling point solvent for PC_61_BM while being a non-solvent for P3HT. The solubilities of P3HT and PC_61_BM in CHO are 0.2 and 23.6 mg/mL, respectively [48]. Results of both studies with films characterized by XPS or grazing-incident X-ray diffraction (GI-XRD) agree that, using this co-solvent approach, higher polymer crystallinities can be observed along with the formation of a P3HT-depleted layer at the surface of the active layer. The addition of 30 vol % of DCM to the active layer solution resulted in an increased PCE in rPSCs of 3.97% as compared to 3.07% for the active layers produced with no solvent additive. In addition to solubility of the active materials, drying velocity of the solvent seems to play a key role in the fabrication of vertically stratified high efficiency P3HT-based rPSCs [49]. Surprisingly, solvent annealing does not give positive effects on the P3HT:PC_61_BM distribution in rPSCs which may be due to the fact that it has to be performed prior to electrode deposition [28].

**Figure 6:**
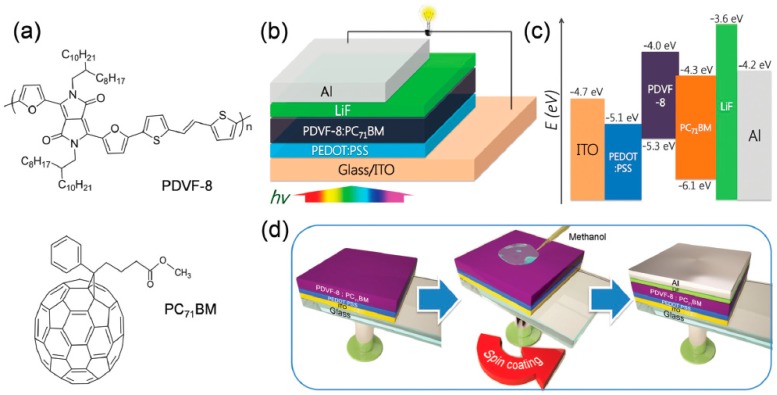
(**a**) Molecular structures of poly{3,6-difuran-2-yl-2,5-di(2-octyldodecyl)-pyrrolo[3,4-c]-pyrrole-1,4-dione-altthienylenevinylene} (PVDF-8) and [6,6]-Phenyl-C_71_-Butyric Acid Methyl Ester (PC_71_BM). Schematic representations of: (**b**) the device architecture; (**c**) the energetic levels and (**d**) the post-deposition surface treatment with methanol. Adapted from [50], with permission from © 2015 Royal Society of Chemistry.

Active layers based on newly introduced polymer donors often use the approach of solvent additives rather than solvent annealing. DIO and 1-chloronaphthalne (CN) are the most utilized solvent additives. In their report, Zhou et al. compared the performances obtained using these two solvent additives during the production of rPSCs active layers composed of poly{3,6-difuran-2-yl-2,5-di(2-octyldodecyl)-pyrrolo[3,4-c]-pyrrole-1,4-dione-altthienylenevinylene} (PDVF-8) and PC_71_BM [50]. Furthermore, they used an additional surface treatment by spin-coating methanol prior to electrode deposition and, consequently, the effects of two solvent processes with active layers prepared in the exact same conditions was observed using XPS depth profiling (Figure 6). The average PCEs measured for their regular architecture devices with no solvent additives is relatively low with an average value of 0.73%. Addition of 3 vol % of DIO and CN increased the PCE to 3.69% and 4.18%, respectively. The additional step of methanol spin-coating on the active layer surface enhanced the PCE of BHJ-rPSCs prepared using CN as a solvent additive up to 4.59%. Their analysis further revealed that as spun active layers (with CN) exhibit a polymer-rich surface and PC_71_BM-rich buried interface which are detrimental to the rPSCs and explain the relatively high Rs and relatively low Rsh obtained in their devices prior to methanol surface treatment. Upon this surface treatment, the vertical concentration gradient is reversed as PC_71_BM molecules diffuse to the surface in contact with methanol. Although not ideal, the resulting vertical concentration profile exhibits slightly more PDVF-8-rich layers close to the bottom interface while PDVF-8-depleted layers are found at the surface of the active layers. Consequently, the Rs in regular device architectures is decreased from 7.7 to 6.2 Ω·cm^2^ upon surface treatment while in the mean time, Rsh increases from 868.7 to 1083.5 Ω·cm^2^ resulting in a higher FF of 60.1% as compared to 58.3% for the untreated device.

**Figure 7:**
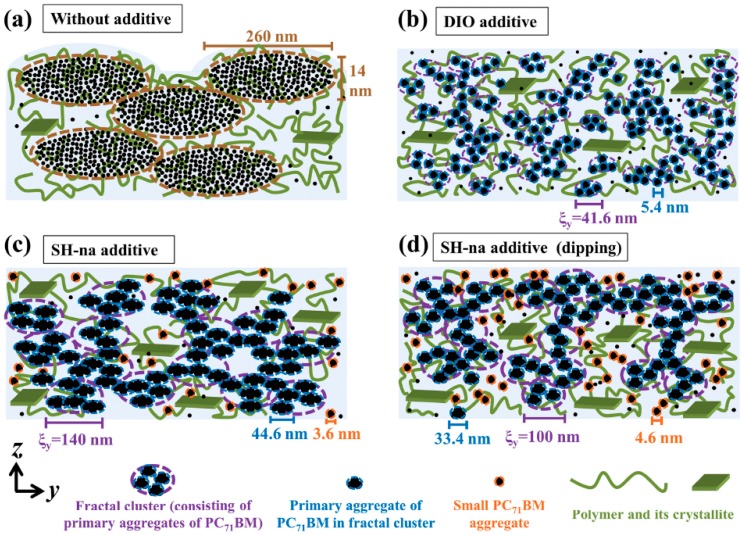
Schematic representation of the active layer morphologies obtained: (**a**) without additive; (**b**) with 1,8-diiodoctane (DIO); (**c**) with 1-Naphthalenethiol (SH-na) and (**d**) with SH-na after solvent treatment. Adapted from [51], with permission from © 2016 WILEY-VCH.

A strategy similar to that mentioned above was also applied to a fluorinated-thieno[3,4-b] thiophene-based polymer (PTB7), which one of the polymers that led the way to overcoming the milestone PCE value of 10% [51]. PTB7:PC_71_BM exhibit higher PCEs when produced using naphthalene-based solvent additives as compared to DIO. In this case, the additive is 1-Naphthalenethiol (SH-na) which leads to PCEs of 7.19% as compared to 3.68% and 6.62% for rPSC devices without solvent additive and with DIO, respectively. The Rs suggests that addition of CN or SH-na does not necessarily lead to formation of the adequate concentration gradient for regular device architectures but rather to a more homogeneous distribution of ED and EA materials throughout the active layer as observed using X-ray scattering combined with atomic force microscopy surface characterizations. The surface treatment, however, clearly increases the amount of PC_71_BM found at the surface of the thin film which explains the enhanced values of FF and PCE obtained in rPSCs after surface treatment by dipping the film in either methanol or methanol containing 4.5 vol % of SH-na. FF and PCE values of 70% and 8.42%, respectively, are obtained for active layer produced with SH-na as solvent after dipping into the methanol containing SH-na solution. Note that the devices prior to dipping have a FF and PCE of 66% and 7.19%, respectively, and that the values for active layers without solvent additives are 46% and 3.68%, respectively. Furthermore, although these results were reported elsewhere, the same active layer surface treated with a mixture of methanol and water resulted in rPSC PCE of 8.14% [52]. The effects of these solvent processes on the resulting active layer morphology are summarized in Figure 7.

For PTB7:PC_71_BM active layers, addition of DIO leads to a homogeneous distribution of ED and EA in the vertical direction. However, results on poly(2,6-Bis(trimethyltin)-4,8-bis(5-(2-ethylhexyl)thiophen-2-yl)benzo[1,2-b:4,5-b′]dithiophene) (PBDTTT-C-T):PC_71_BM active layers prepared using DIO as solvent additive suggest otherwise [53]. The two polymers have relatively similar conjugated backbones with different substituents which easily explains the formation of different vertical profiles. XPS measurements reveal that in PBDTTT-C-T:PC_71_BM active layers deposited from DCB, surfaces containing a 1:0.88 ED:EA ratio are formed. Upon addition of DIO, this ratio is increased to 1:0.48, indicating that a polymer-rich layer is formed at the active layer surface, which should be beneficial for iPSCs. In fact, these observations are well correlated with the device performances, especially in the case of those prepared using DIO (Table 2).

As suggested by Figure 7b, the addition of DIO to the blend solution results in the formation of smaller phase separated domains which enhance the Jsc and FF (easier percolation and increased crystallinities) of the devices. Upon addition of DIO, Jsc and FF increase 16% and 25%, respectively, for rPSCs while in the case of iPSCs, these parameters increase 25% and 49%, respectively. The much larger increases observed for iPSCs are a direct consequence of the formation of a polymer-rich layer at the interface with the top anode in the inverted architecture devices. The improved morphology in both horizontal and vertical directions in iPSCs consequently lead to the fabrication of device displaying PCEs up to 9.13% with solvent additives as compared to 5.19% for the active layers deposited from DCB [53]. Note that, addition of DIO decreases the Voc in both rPSC and iPSC. This may be related to changes in the HOMO level of PBDTTT-C-T upon crystallization. In fact, the authors observed variations in surface potential of the active layers upon addition of DIO.

In summary, when it comes to solvent processes, the use of solvent additives and post-deposition surface treatments seem to be more efficient compared to solvent annealing. As mentioned previously, this may be also related to the fact that, as solvent annealing requires to be performed prior to electrode deposition, the surface of the active layers during solvent annealing are almost always in contact with air. However, Liu et al. introduce an elegant method to undergo solvent annealing while the active layer surface is in contact with another material, namely, poly(dimethylsiloxane) (PDMS) [54]. Using PCDTBT:PC_71_BM active layers for rPSCs, the authors compare the performances of pristine, solvent annealed with a mixture of tetrahydrofuran and carbon disulfide, and solvent annealed with PDMS deposited on top active layers for PSCs. Using XPS, the authors demonstrate that the pristine active layers exhibit a polymer-rich surface with a ED:EA ratio of 4:1. Upon mixed solvent annealing, this ratio is decreased to 3:1 and solvent annealing with PDMS deposited on top of the active layer leads to a ratio of 2:1. These results confirm that PC_71_BM gradually diffuse to the surface when solvent annealing is used on this ED–EA system. Note that the solvents used for solvent annealing in this case are rather different than those commonly used for solvent annealing. However, the diffusion of PC_71_BM molecules is well correlated with the increase in Jsc and FF of rPSCs. Jsc for the pristine, solvent annealed and solvent annealed with PDMS active layers are 10.80, 11.92 and 12.03 mA/cm^2^, respectively, while the FF evolves from 49% to 62% and 64%, respectively. Additionally, the Rs value gradually decreases from 15.98 to 7.66 Ω·cm^2^ while the Rsh increases from 1060 to 2322 Ω·cm^2^ indicating that leak current is extremely reduced in those regular architecture devices. The Voc is only mildly affected with a small increase from 0.88 V for pristine and solvent annealed active layers to 0.89 V when using PDMS-assisted solvent annealing. It is worth mentioning here that this approach may not be used with all materials combination. A large number of interactions have to be taken into account to obtain the ideal vertical profiles in active layers for PSCs [14]. As we will discuss in the following section, minor chemical structure modifications of the active materials can have major effects on the formation of adequate vertical distributions. Note that surface-enrichment with either ED or EA is not limited to solvent vapors but can also be achieved using gases such as CO_2_ which strongly interacts with PC_61_BM molecules [55].

### 2.3. Chemical Modification of Active Materials to Induce Adequte Vertical Distribution

The chemical nature of the active materials influences their interactions with each other, with the solvent used for active layer deposition and with the substrate on which they are deposited [14]. In fact, comparing results obtained using PCDTBT and its –OCH3 di-substituted derivative (PCDTBT1), one can already see the influence of small modifications on the vertical concentration gradients and the consequent effect on device performances [38,40]. In the two references above, the pristine films deposited on PEDOT:PSS show opposite trends with PCDTBT:PC_71_BM active layers displaying a polymer-rich surface while PCDTBT1:PC_71_BM thin films exhibit a more adequate vertical distribution for rPSC with a PC_71_BM-depleted layer at the buried interface with PEDOT:PSS. Note that these two studies use different ED:EA ratios which may also influence the vertical ED:EA concentration gradient in the thin films [56,57]. Similarly, the molecular weight (Mw) of the polymer may affect the vertical profiles and thus to adequately compare results and PSC performances in the remaining of this section, we will focus on studies directly comparing the influence of chemical modification of the ED or EA materials [58].

**Figure 8:**
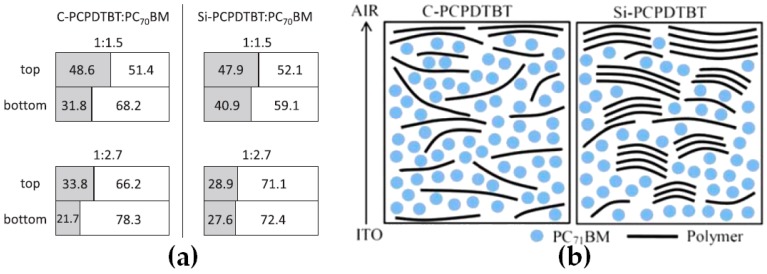
Schematic representations of: (**a**) the top and bottom layer compositions and (**b**) the vertical distribution of donor and acceptor molecules in poly[2,1,3-benzothiadiazole-4,7-diyl[4,4-bis(2-ethylhexyl)-4H-cyclopenta[2,1-b:3,4-b′]dithiophene-2,6-diyl]] (C-PCPDTBT) and poly[2,1,3-benzothiadiazole-4,7-diyl[4,4-bis(2-ethylhexyl)-4H-cyclopenta [2,1-b:3,4-b′]dithiophenesiloe2,6-diyl]] (Si-PCPDTBT)-based active layers. Adapted from [56,59], with permission from © 2014 Elsevier and © 2014 WILEY-VCH.

Dithiophene-based polymers such as poly[2,1,3-benzothiadiazole-4,7-diyl[4,4-bis(2-ethylhexyl) -4H-cyclopenta[2,1-b:3,4-b′]dithiophene-2,6-diyl]] (C-PCPDTBT) and poly[2,1,3-benzothiadiazole-4,7-diyl[4,4-bis(2-ethylhexyl)-4H-cyclopenta[2,1-b:3,4-b′]dithiophenesiloe2,6-diyl]] (Si-PCPDTBT) provide the ideal framework to observe how small modifications of the chemical structures can influence the vertical concentration gradients. In this particular case, the modification consists in replacing the carbon bridging atom in C-PCPDTBT with a silicon atom in Si-PCPDTBT [56,59]. Using SE, Georgiou et al. studied the two polymers blended with PC_71_BM with the objective of selecting the adequate material combination for rPSC fabrication [56]. Their results emphasize that in C-PCPDTBT:PC_71_BM active layers, polymer-rich surfaces and fullerene-rich buried interfaces can be observed. This vertical concentration profile is particularly detrimental for rPSCs and by changing the bridging atom to silicon, they observed that a more homogeneous ED:EA distribution can be found in Si-PCPDTBT:PC_71_BM thin films (Figure 8a). Unfortunately, the device performances reported by the authors were limited to the Si-PCPDTBT:PC_71_BM active layers which does not allow to correlate their observations on vertical profiles with bridging atom dependent device performances. On the other hand, in the study presented by Lin et al., active layers using the same polymer:fullerene combinations were produced to fabricate iPSCs [59]. Similar vertical concentration gradients were observed using XPS depth profiles with a polymer-rich top layer obtained in C-PCPDTBT:PC_71_BM active layers while, upon substitution of the bridging atom with silicon, a more homogeneous vertical ED:EA distribution is generated (Figure 8b). In iPSCs, a polymer-rich surface and fullerene-rich buried interface can be favorable for efficient charge extraction. Accordingly, inverted devices prepared with C-PCPDTBT, which display such concentration gradient, exhibit higher performances (PCE of 3.89%) as compared to those using Si-PCPDTBT:PC_71_BM active layers (PCE of 3.17%). Note that modifying the structure is not limited to bridging atoms and synthesis of copolymers consisting of conjugated and electrically insulating blocks or functionalizing the polymer also provide means to tune the vertical concentration gradients in PSC active layers [60,61].

**Figure 9:**
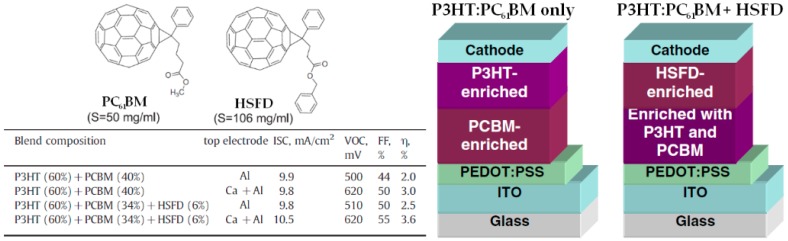
Molecular structures, device parameters and schematic representation of the effect of highly soluble fullerene derivative (HSFD) addition to P3HT:PC_61_BM active layers for rPSCs. Adapted from [63], with permission from © 2010 Elsevier.

Although recent advances in materials research have introduced the use of other EAs in PSCs, the state-of-the-art EAs are still considered to be fullerene derivatives [6]. Among those, the most commonly used ones are PC_61_BM and PC_71_BM. Due to their difference in solubility in chlorobenzene (CB), we verified that different vertical concentration distribution can be obtained in regular device active layers composed of a high efficiency naphthobisthiadiazole-based polymer (PNTz4T [2]) with either PC_61_BM or PC_71_BM. While the overall concentration gradient observed using EDS is not affected remarkably, the use of PC_71_BM, which has a lower solubility in CB, lead to the formation of alternating polymer-rich/fullerene-rich layers [62]. This suggests that even minor changes in the chemical structure of the fullerene derivative could have major effects on the vertical composition in the thin films. For instance, replacing PC_61_BM with its fluorinated analog (FPCBM) in P3HT:fullerene thin films resulted in large changes in ED:EA compositions at the top and bottom of the active layers especially when prepared on Cs_2_CO_3_-modified ITO substrates for iPSC fabrication [26]. The fullerene/P3HT ratio at the buried interface calculated using XPS increased from 7.73 for PC_61_BM to 10.88 for FPCBM when the active layers are deposited using a high spin-coating speed of 3000 rpm followed by thermal annealing at 110 °C for 10 min. In the iPSCs, upon replacing PC_61_BM with FPCBM, FF and PCE were enhanced from 40.59% to 55.69% and from 1.93% to 2.70%, respectively. A similar approach was used by developing another fullerene derivative (HSFD, Figure 9) with a very different solubility in CB as compared to PC_61_BM [63]. The solubilities for PC_61_BM and HSFD in CB were experimentally determined to be 50 and 106 mg/mL, respectively. While the P3HT:PC_61_BM only active layer displays a vertical distribution detrimental to rPSCs, by replacing a small amount of PC_61_BM with HSFD, a fullerene-rich top layer is generated resulting in large increases in Voc, FF and PCE (Figure 9).

**Figure 10:**
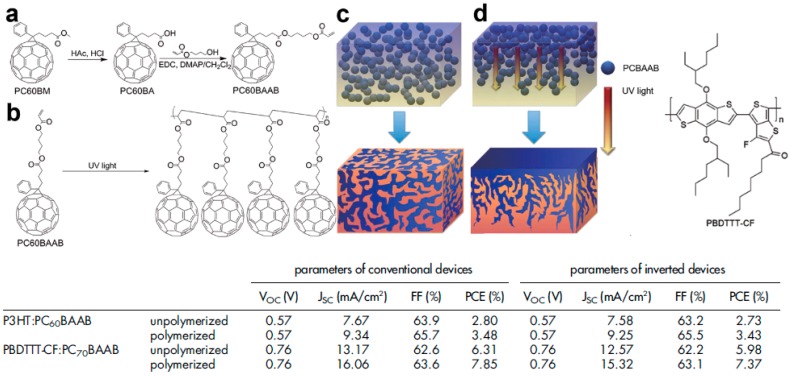
(**a**) Synthetic route of photo-polymerizable fullerene monomers; (**b**) photo-polymerization of the monomer and (**c**,**d**) schematic representations of active layers prepared using (**c**) conventional annealing processes and (**d**) photo-polymerization along with the chemical structure of the polymer donor and the device performances. Adapted from [64], with permission from © 2014 Nature Publishing Group.

The fullerene derivatives can also be functionalized to generate photo-polymerizable EAs [64]. This elegant approach to generate vertical concentration gradients by in-situ polymerization introduced by Zhang et al. requires a fair amount of chemistry, but the results as well as the process to generate vertical distribution are rather impressive. The authors developed a process which can produce the adequate vertical morphology in active layer for either rPSCs or iPSCs by simply changing the side from which they shine light to induce photo-polymerization. In fact, in PSCs active layers, due to the absorption from the active molecules, a light-absorption gradient is produced. This light-absorption gradient has been previously used to induce vertical donor–acceptor distributions favorable for iPSCs [65]. Using XPS, the authors verified that by shining light from either the top or the bottom side of the active layers, the fullerene derivative (PCBAAB) diffuse in the direction opposite to the light which results in the possibility to form adequate vertical concentration gradients for both regular and inverted device architectures starting from the same material blend (Figure 10). For P3HT:PCBAAB active layers, the PCE is increased from approximately 2.75% for unpolymerized films to 3.48% and 3.43%, respectively for rPSCs (photopolymerized from the bottom side) and iPSCs (photopolymerized from the top side). Similar improvements were obtained using a higher performance polymer paired with the C_70_-derivative of PCBAAB and PCEs up to 7.37% and 7.85% were reported for iPSCs and rPSCs, respectively.

In summary, numerous methods have been proposed to tune the vertical concentration gradients in the active layers of PSCs which would be adequate for either regular or inverted device architectures. Some of these approaches are relatively simple and consist of thermal or solvent annealing, solvent additives or surface treatment using solvent and gases. More advanced solutions have also been proposed which are based on chemical modification of either the ED (conjugated polymer) or EA (fullerene derivative) materials. The interfacial forces with the substrate and at the top surface play an essential role in the formation of the adequate vertical concentration gradients. These studies were all based on single layer active layers but another approach is the formation of multilayer active layers in which each layer contains a different ED:EA ratio. The issue then becomes that of depositing additional layers without damaging the underlying ones.

## 3. Sequential Deposition Processes to Fabricate Multilayer Active Layer PSCs

As ED and EA molecules are soluble in only a small number of solvents (usually chlorinated solvents), producing multilayer active layers represents a technological challenge which gave rise to some innovative PSC fabrication processes. While some of these processes such as floating of active layer films resulted in relatively low PCEs [66], the works presented below demonstrate that generation of multilayer active layers can successfully be achieved and generally results in large increases in PCEs either by sequentially depositing multilayers with various ED:EA concentrations or by adding ED or EA buffer layers to the device architectures. As fullerene derivatives are in general more soluble in solvents used for PSC active layer fabrication, the fabrication of sequentially deposited multilayers for iPSCs, in which the bottom layer should be highly concentrated in fullerene derivatives, becomes even more difficult.

### 3.1. Fabrication Processes to Generate Inverted Multilayer Active Layers

While solubility of PC_61_BM and PC_71_BM in solvents such as CB or DCB is relatively high, the fullerene molecules themselves (C_60_ or C_70_) are almost insoluble in most solvents. Consequently, they are not solution-processable materials but still provide a clear method to verify whether the formation of multilayers for iPSCs could be an efficient approach to fabricate high PCE devices. Chang et al. evaporated C_70_ fullerene molecules onto the cathode substrate followed by high-speed (60 to 350 mm·s^−1^) blade coating of PBDTTT-C-T or PTB7 from a mixture of toluene and *o*-xylene [67]. Using this fabrication process, the authors were able to develop iPSCs with PCE up to 6.55% and 7.15%, respectively for PBDTTT-C-T and PTB7 produced with no solvent additives. While these device performances are accompanied by high FF over 65%, the use of evaporated molecules as buffer layers results in a long and energy-consuming process (necessity for two separate vacuum steps for the bottom buffer layer and top electrode). Very interestingly, from the transmission electron microscopy cross section of the active layers, the authors concluded that the two-step active layer fabrication process leads to the formation of DfBLs in which the top polymer layers partially diffuse into the underlying C_70_ layer to generate intermixed layers sandwiched between pure C_70_ and polymer buffer layers for iPSCs. As discussed in Section 3.3, developing such trilayer active layers through a two-step sequential deposition process may be an extremely interesting approach to generate high performance PSCs. Note that, although C_70_ should be entirely insoluble in the used solvent mixture, it is safe to assume that part of the deposited C_70_ is washed-off during the top layer deposition. To avoid this wash-off phenomenon upon top layer deposition, a second innovative approach was developed in which benzoic acid substituted C_60_ molecules (C_60_-SAM) were covalently bond to the substrate acting as self-assembled buffer layers [68]. Depositing P3HT:PC_61_BM blends on top of the tightly bond C_60_-SAM layer generates EA/ED:EA BHJ double layer active layers. The device PCE increased from 2.8% to 3.8% upon addition of the C_60_-SAM layer. In fact, all photovoltaic parameters were improved with a particularly large enhancement of FF from 49.6% to 57.2% associated with a decrease in Rs from 13 to 2.4 Ω·cm^2^ and an increase in Rsh from 380 to 1010 Ω·cm^2^.

**Figure 11:**
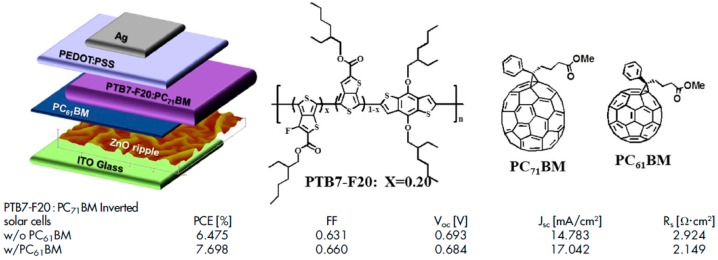
Device architecture, molecular structures and device parameters of sequentially deposited bilayers for inverted polymer solar cells. Adapted from [69], with permission from © 2014 Nature Publishing Group.

These two approaches rely on either energy (thermal evaporation of C_70_) or time-consuming (synthesis of C_60_-SAM and grafting of the monolayer) processes which should be avoided, especially when potential industrial applications are involved. Consequently, a process based on sacrificial solvent spin-coating prior to top layer deposition was developed to generate similar sequentially deposited active layers [69]. In this study, a BHJ composed of a PTB7 derivative (PTB7-F20) and PC_71_BM is deposited on top of a PC_61_BM buffer layer (Figure 11). The additional PC_61_BM layer inserted at the interface between the BHJ and the ZnO-covered ITO substrates results in a large increase in Jsc, FF and consequently, PCE. Although the authors do not show any clear evidence that the underlying PC_61_BM remains unwashed, by spin-coating ethanol immediately followed by BHJ deposition, it is suggested that no washing off or mixing occurs during the process. The increase in FF from 63.1% to 66.0% along with the enhancement of Jsc from 14.783 to 17.042 mA/cm^2^ with the additional fullerene derivative layer suggest that the underlying does indeed, at least partially, remain unwashed. Similar results (enhancement of PCE upon additional fullerene buffer layer) have been observed on the P3HT:PC_61_BM active layers where no sacrificial solvent was used [70]. The strategy used for BHJ deposition on top of the fullerene buffer layer in this study was to anneal the buffer layer at 150 °C for 10 min prior to subsequent layer deposition to induce the formation of crystalline PC_61_BM displaying increased resistance to organic solvents. Here again, the amount of washing-off that occurs during the process was not investigated but the Jsc, Voc and FF all exhibit large enhancements leading to an increase from 3.39% to 4.50% of the PCE upon insertion of the additional PC_61_BM layer. In fact, the performances were further increased to 4.97% by laminating a glass substrate covered with the anode and a thin P3HT film to finalize the device instead of directly evaporating the anode materials on top of the active layer.

**Figure 12:**
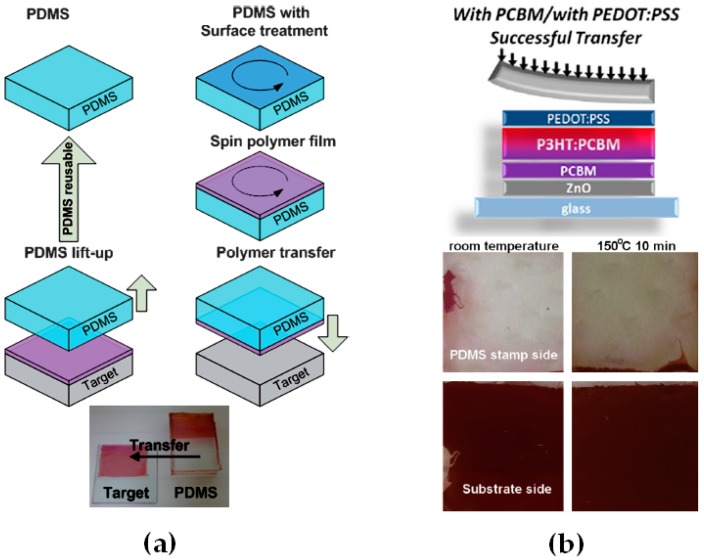
Schematic representation and transfer efficiency of transfer-printing processes using poly(dimethylsiloxane) (PDMS) surface modification with: (**a**) solvent spin-coating; and (**b**) poly(3,4-ethylenedioxythiophene) polystyrene sulfonate (PEDOT:PSS) interlayer insertion. Adapted from [74,75], with permission from © 2009 Royal Society of Chemistry and © 2016 Taylor & Francis.

Lamination is a dry-transfer process which ensures that two parts of the active layer (e.g., polymer-concentrated and fullerene-concentrated ones or pure P3HT and PC_61_BM layers) can be assembled without mixing between the two layers [70,71]. While lamination itself only allows for the formation of bilayer active layers, the use of micro-contact-printing or transfer-printing consists of alternative dry-transfer processes to generate multilayer active layers without any mixing between two sequentially deposited layers [72,73,74,75]. The two main issues for efficient transfer-printing are to deposit high quality films from chlorinated solvents on the substrates used as stamps and be able to entirely transfer the films from the stamp to the device substrate. The most commonly used substrate for transfer-printing is PDMS which not only has low wettability properties but is also easily swollen by chlorinated organic solvents such as chloroform (CF), CB and DCB. To generate high quality P3HT, PC_61_BM or blend films on PDMS, Huang et al. developed a method based on surface treatment of PDMS with CF prior to deposition of the films from solutions in CF (Figure 12a) [74]. This approach led to the fabrication of PCBM/P3HT bilayers in iPSCs with relatively low FF and PCE of 34.2% and 0.97%, respectively. Upon thermal annealing at 160 °C, the FF and PCE increase to 59.6% and 2.83%, respectively. The authors attribute this enhancement to the better contact between the two deposited layers resulting from the reorganization of polymer chains when temperatures above 160 °C are used. Taking into account the discussion in the first part of this review, it is safe to assume that PC_61_BM may also diffuse through the film to form intermixed layers located between P3HT and PC_61_BM buffer layers. An alternative controlled transfer process was more recently proposed to generate adequate vertical concentration profiles in transfer-processed bilayer active layers for iPSCs [75]. Unlike the previous study, PDMS was first covered with a PEDOT:PSS layer (which acts as a hole only layer in iPSCs) and then with a P3HT:PC_61_BM BHJ. The two layers were then simultaneously transferred onto a PC_61_BM-covered transparent cathode and the devices were finalized by anode evaporation. The PEDOT:PSS and PCBM interlayers not only act as hole and electron only layers, respectively, but through work of adhesion calculations, these two interlayers proved to be essential for successful transfer and fabrication of multilayer active layers for inverted devices. The transferred devices exhibit an increase in FF and PCE of approximately 40% with respect to the spin-coated reference devices. These large enhancements were attributed to the formation of an adequate vertical concentration profile observed by GI-XRD with P3HT-rich and PC_61_BM-rich layers, respectively, at the top and bottom of the bilayer active layer. While the PC_61_BM-rich layer results directly from the fabrication process, the P3HT concentrated top layer is a consequence of the diffusion of PC_61_BM molecules from the blend to the bottom interface with the PC_61_BM-only layer during the transfer process performed at 150 °C.

### 3.2. Fabrication Processes to Generate Regular Multilayer Active Layers

**Figure 13:**
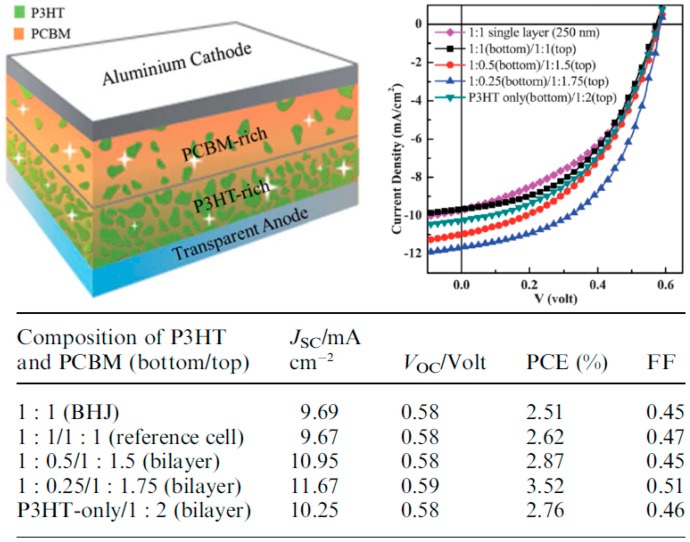
Schematic representation, typical J–V curves and photovoltaic device parameters of multilayer transfer-printed regular architecture devices. Adapted from [77], with permission from © 2012 Royal Society of Chemistry.

Similar transfer-printing strategies have also been used for the fabrication of multilayer active layer rPSCs either with PDMS or with other stamp materials [74,76,77]. In fact, using ultraviolet curable polycarbonate films for transfer-printing, Wang et al. fabricated flat bilayer devices (P3HT/PC_61_BM) which, upon thermal annealing generate an intermixed layer [76]. The resulting devices exhibit a PCE of 3.24%, displaying an increase with respect to the single BHJ layer devices which have a PCE of 2.85%. Similarly to single BHJ active layers, vertical ED–EA distribution in P3HT/PC_61_BM bilayers which were initially planar can be modified through thermal annealing induced interdiffusion of the molecules [66]. Research on sequential transfer-printing of multilayer active layers was further advanced through a systematic study from Kuo et al., in which the ED:EA ratios of the bottom and top layers were tuned to generate the highest possible performances in rPSCs (Figure 13) [77]. The device parameters clearly demonstrate that the performances increase with increasing concentration of P3HT and PC_61_BM in the bottom and top layers, respectively. In particular, the devices with 1:0.25 (bottom)/1:1.75 (top) ED:EA ratios exhibit high FF and PCE of 51% and 3.52%, respectively, while those of the transferred BHJ (1:1 ratio) only display values of 45 and 2.51%. It is worth mentioning that the insertion of a P3HT-only buffer layer on top of which a BHJ (1:2 ratio) was transferred also result in small increases with respect to the reference cells. XPS depth profiles were employed to verify that the fabricated active layers actually exhibit the adequate vertical concentration gradient.

Although transfer-printing seems to be a powerful process to deposit BHJ on top of P3HT buffer layers, much higher PCE enhancement were obtained by direct solution processes for rPSCs. In fact, deposition of non-soluble molecules such as thiophene or covalent bonding of a derivative of the polymer ED resulted in large improvements of the photovoltaic parameters of rPSCs [78,79]. For example, covalently bonding a fluorinated derivative of PBDTTT-C (PBDTTT-CF) to the bottom anode resulted in a FF increase from 64.1% to 67.9% and a PCE enhancement from 6.2% to 7.1% in comparison with the devices deposited directly on the anode substrate [79]. In the case of high crystallinity polymers such as P3HT, the formation of a bottom polymer-only layer can be obtained much more simply as they are less soluble in the solvents used for BHJ deposition. Taking into account this lower solubility (as compared to fullerene derivatives), using a more crystalline high Mw P3HT should lead to the formation of a P3HT buffer layer which will not be entirely washed out upon deposition of a top BHJ layer. Using this strategy, Liang et al. prepared P3HT:PC_61_BM BHJ devices with or without a 15 nm thick P3HT buffer layer at the PEDOT:PSS/BHJ interface [80]. The Jsc and FF of the reference device (BHJ only), which were 9.78 mA·cm^−2^ and 67.8%, respectively, were increased to 12.00 mA·cm^−2^ and 69.0% for the P3HT-only/BHJ bilayer device. The authors also emphasize that these results can only be obtained with high Mw P3HT (less soluble in organic solvents) as using low Mw P3HT results in complete washing off of the buffer layer.

**Figure 14:**
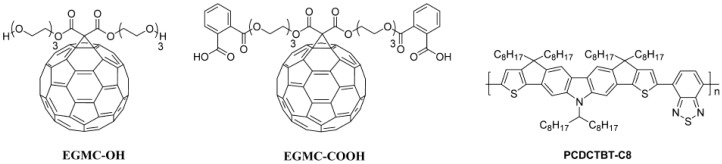
Molecular structures of active materials used in alcohol/H_2_O soluble fullerene derivative top layers for regular device architectures. Adapted from [85], with permission from © 2013 American Chemical Society.

To avoid washing off the underlying layers, the simplest method consists in depositing the top layers from non-solvents for the materials deposited first. For instance, deposition of multilayers with various P3HT:PC_61_BM ratios was achieved by using water-based nanoparticles deposited sequentially [81]. However, this elegant and eco-friendly approach led to poor device performances with a maximum PCE of 0.457% which is much lower than that obtained using conventional BHJ deposition techniques (2.34%). In fact, for rPSCs, evaporation of a top C_60_, C_70_ or PC_61_BM was also considered as, unlike iPSCs, these can be evaporated immediately before electrode deposition [82,83]. In other words, there is no necessity for the generation of an additional high vacuum step. This strategy was used to produce bilayers of a cyclopentadithiophene based polymer covered with C_70_ which exhibited, after both pre- and post-annealing processes at 200 °C for 1 h each, a PCE of 2.85% [82]. On the other hand, evaporation of a PC_61_BM buffer layer on top of a P3HT:PC_61_BM BHJ proved to be an efficient method to enhance the electron collection in rPSCs [83]. In particular, when a 7 nm-thick buffer layer is deposited, the PCE of the devices increases from 3.8% to 4.5%. While these approaches use evaporated molecules, due to difference in solubility between P3HT and PC_61_BM with respect to some solvents, all-solution-processed multilayer active layers can be successfully formed by using orthogonal solvents for BHJ and top buffer layer [84]. In fact, solvents such as CHO or DCM could provide the adequate properties to generate a top PC_61_BM buffer layer as the solubility of P3HT in those two solvents is extremely low. Note that BHJ composed of polymer and fullerene derivatives usually correspond to the fullerene molecules dispersed in the polymer matrix. Using CHO or DCM could however result in partial dissolution of the PC_61_BM molecules in the BHJ during the top layer deposition. Nonetheless, Tremolet de Villers et al. proved that by depositing a top PC_61_BM layer from DCM solutions to cover the P3HT:PC_61_BM BHJ, much more reproducible device performances can be obtained due to a more efficient charge collection at the active layer/cathode interface [84]. In order to completely remove the potential dissolution of PC_61_BM molecules in the underlying BHJ layer, Lai et al. synthesized water and alcohol-soluble C_60_ derivatives (EGMC-OH and EGMC-COOH, structures presented in Figure 14) which can be deposited as a top buffer layer and further doped with alkali carbonates [85].

Their results for PCDCTBT-C8 (Figure 14):PC_71_BM/fullerene derivative bilayer rPSCs are summarized in Table 3. The performances displayed in Table 3 were obtained using top layers prepared by dissolving either EGMC-OH or EGMC-COOH in a 10:1 mixture of 2-ethoxyethanol:H_2_O. Using this solvent mixture ensures that the underlying layer remains entirely unwashed. However, the addition of undoped top fullerene layers only mildly increase the device performances through the Jsc enhanced from 9.02 to 9.43 and 9.61 mA·cm^−2^, respectively, for the BHJ only, top EGMC-OH and top EGMC-COOH devices. Upon doping of the additional top EGMC-COOH layer with Li_2_CO_3_, the PCE increased from 3.61% for the single BHJ rPSC to 4.29% for the bilayer devices.

Note that similar device performances have been obtained with undoped polyethylene glycol substituted fullerenes (PEG-C_60_) top buffer layers with PCEs reaching 3.84% [86]. These top layers were deposited from DCM which, according to the authors, does not dissolve P3HT. Based on this difference in solubility and the limited diffusion of PEG-C_60_ molecules into the P3HT network, the authors generated DfBL active layers in which the thin film surface is highly concentrated in self-assembled PEG-C_60_ molecules acting as an electron only buffer layer and the device PCE is increased to 4.40%. In fact, the DfBL approach has become increasingly popular since 2009 and in the following section we will discuss whether DfBL PSCs have the potential to overcome BHJ PSCs.

### 3.3. DfBL Active Layers for Regular Device Architectures: Alternative Deposition Process with the Potential to Overcome BHJ PSC Performances

**Figure 15:**
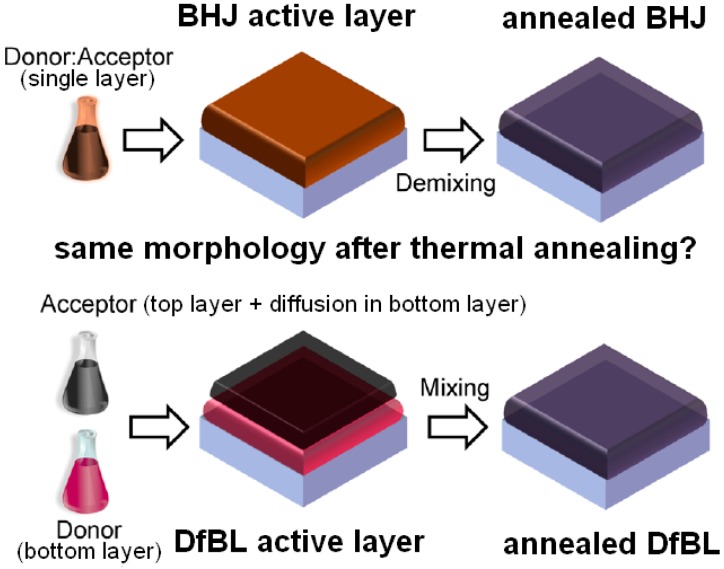
Schematic representation of the fabrication of bulk heterojunction (BHJ) and diffusive bilayer (DfBL) active layers as well as the debate on their post-annealing morphology. Adapted from [99], with permission from © 2014 American Chemical Society.

The concept of DfBL PSCs was brought to light by a meticulous study undergone by Ayzner et al. in 2009 [87]. In DfBL PSCs, active layers consist of sequentially deposited polymer ED:fullerene derivative EA bilayers (Figure 15). However, the objective of this strategy is not to form planar heterojunction but to allow for the fullerene derivative to penetrate and gradually diffuse inside the underlying polymer network. For instance, P3HT has a very low solubility in DCM but the solvent can swell the P3HT film. By depositing a PC_61_BM solution in DCM on top of the P3HT layer, Ayzner et al. demonstrated that devices with PCEs up to 3.5% can be produced. This approach has since been applied to other ED:EA systems attempting to increase the device performances [88,89,90,91,92,93,94,95,96]. As a result, DfBL PSCs now exhibit PCEs higher than 8% [96]. Nevertheless, the question of whether the obtained active layer morphologies are equivalent or significantly different as compared to the conventional BHJ PSC active layers is still under debate [88,97,98,99,100,101,102]. The initial studies stipulated that the two deposition processes were equivalent in terms of resulting active layer morphologies, especially after thermal annealing when characterized using neutron reflectometry (Figure 15) [97,98,99]. More recently, other works have demonstrated that a straightforward answer may not be easily obtained and that parameters such as solvent polarity, volatility as well as polymer crystallinity may play essential roles in the formation of ideal vertical concentration gradients in DfBL active layers [93,100].

**Figure 16:**
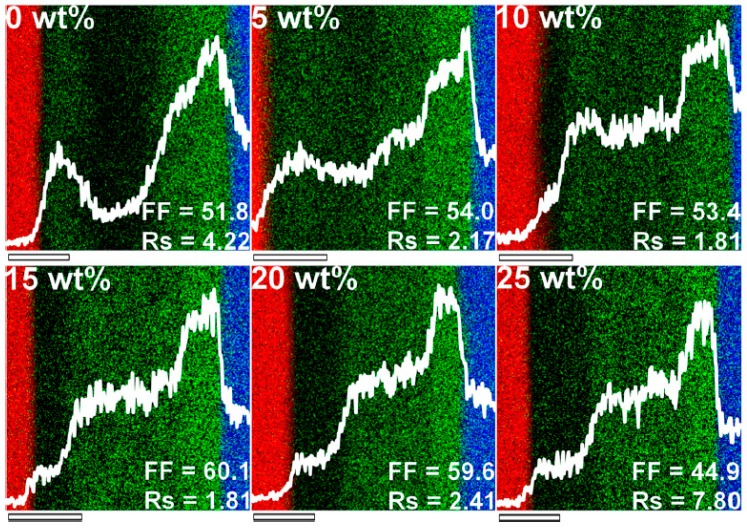
Regiorandom poly(3-hexylthiophene) addition effect on the formation of adequate vertical concentration gradients, fill factor (FF) and series resistance (Rs) of annealed diffusive bilayer active layers. The blue, green and red signals correspond to In, S and Al, respectively, and the white lines are the S profiles obtained throughout the device cross-sections. The scale bars correspond to 50 nm. Adapted from [103], with permission from © 2012 American Institute of Physics.

Consequently, increasing or decreasing the polymer crystallinity may be a good strategy to generate the ideal vertical ED–EA distribution in DfBL rPSCs. In fact, we demonstrated using EDS that adding regiorandom P3HT (RRa-P3HT) to the commonly used regioregular P3HT in the first deposited layer allows for an increased control over the vertical morphology of P3HT:PC_61_BM DfBL PSCs after thermal annealing treatment [103]. Most studies agree that, while as deposited DfBL active layers exhibit the ideal vertical concentration gradients, this positive profile is lost upon thermal annealing as P3HT crystallizes leading to redistribution of PC_61_BM molecules in the thin film [97]. Here, the low crystallinity of RRa-P3HT limits the rearrangement upon thermal annealing and we can observe post-annealing concentration graded vertical profile formation with increasing RRa-P3HT concentration (Figure 16).

As a result, the device FF largely increases with RRa-P3HT concentration up to 15 wt %. As low P3HT crystallinity also leads to lower hole conduction properties, upon addition of more than 15 wt %, the device FF and performances gradually decrease. On the other hand, if P3HT is already in a highly crystalline state prior to PC_61_BM deposition, the effect of thermal annealing on the vertical concentration gradient of already crystalline P3HT and PC_61_BM molecules will be limited. Hence, increasing the P3HT crystallinity either by using mechanical processes or through solvent additives similarly led to large PCE increases (over 30% with respect to conventional devices) in DfBL PSCs [104,105,106]. The possibility to individually tune each layer in P3HT:PC_61_BM DfBL active layers also represents a major advantage to improve device performances by introducing methods and processes which do not have a positive effect in BHJ-PSCs such as nanostructuration of P3HT film surface to obtain a larger ED–EA interface or surface treatment and doping of the p-type polymer layer for enhanced hole conductivities [107,108,109,110]. The highest PCE for P3HT:PC_61_BM DfBL-PSCs was in fact obtained for doped P3HT layers leading to efficiencies of approximately 4% [109]. Although this value is fairly high for the P3HT:PC_61_BM ED:EA pair, higher performance BHJ-PSCs can be produced. On the other hand, when it comes to some recently introduced high performance polymers, DfBL often overcome the PCEs obtained for BHJ-PSCs [94]. For instance, in BHJ-rPSCs, PBDTTT-C-T:PC_61_BM active layers barely reach PCEs of 4.5%, while DfBL-PSCs easily display values over 6%.

Some of the low-bandgap polymers have a relatively amorphous nature (low crystallinity) and therefore, become soluble in DCM. In consequence, the DfBL active layer formation method cannot be used in a straightforward manner because the polymer layer is washed off during the top layer deposition. For instance, this is the case for PCDTBT [95]. However, using EDS and AFM cross-sections, Seok et al. demonstrated that the adequate use of solvent additives can generate extremely positive results provided that the bottom PCDTBT layer is not washed-off during the process and that high quality PC_71_BM layers can be deposited. The device performances of the PCDTBT:PC_71_BM DfBL-PSCs processed without any solvent additives are relatively low with PCEs of approximately 1.1%. Upon addition of DIO to the bottom PCDTBT layer, the PCE is improved to 1.8% as the high boiling point solvent additive leads to better molecular arrangement and consequently, an increase in resistance of the PCDTBT layer to DCM. Using this approach, relatively low performances are obtained as compared to their BHJ-PSCs equivalents. This was attributed to poor quality PC_71_BM layer formation as a result of the low solubility of the fullerene derivative in DCM. To overcome this issue, a second solvent additive (diiodomethane, DIM) was used to increase the fullerene concentration in the solution and process high quality top PC_71_BM layers. The combination of these two strategies resulted in a large improvement of the photovoltaic parameters up to 7.1% which further confirms that being able to independently process the bottom ED and top EA layers in DfBL can become a great advantage for some polymer:fullerene derivative systems (Table 4).

**Figure 17:**
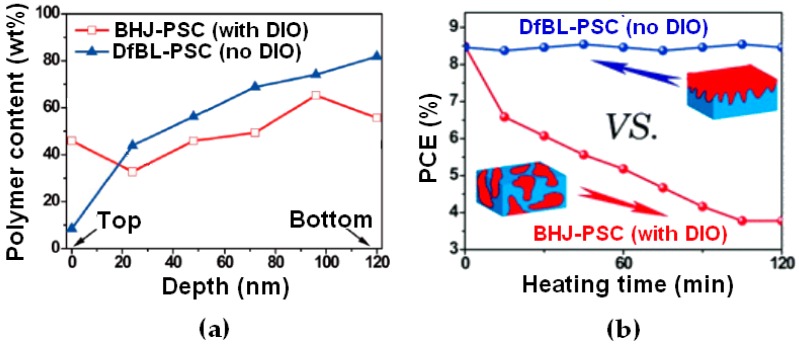
(**a**) Vertical electron donor-electron acceptor profiles and (**b**) thermal stability tests of fluorinated-thieno[3,4-b] thiophene-based polymer:[6,6]-Phenyl-C_71_-Butyric Acid Methyl Ester (PTB7-Th:PC_71_BM) diffusive bilayer- and bulk heterojunction-polymer solar cells. Adapted from [96], with permission from © 2016 Royal Society of Chemistry.

While the results obtained using solvent additives in PCDTBT:PC_71_BM DfBL-PSCs are extremely positive, using solvent additives often results in decreased stability of the device in the long term [111]. Consequently, avoiding the use of solvent additives can be a major advantage for potential industrial applications, as it not only removes the formulating step of the various solutions but could also produce long-lasting devices. Using a PTB7 derivative (PTB7-Th) and PC_71_BM, Cheng et al. demonstrated that similar PCEs of 8.3% and 8.5% can be obtained for both BHJ- and DfBL-PSCs based on this ED–EA combination, respectively [96]. However, although the PCEs are very similar, there are major differences for the two types of active layer in terms of vertical concentration gradient and thermal stability (Figure 17, data calculated from X-ray scattering measurements). The best performing DfBL devices are obtained without the use of any solvent additives while the best BHJ-PSCs use additional DIO. In Figure 17a, the adequate polymer vertical distribution for rPSCs is formed in DfBL-PSCs while BHJ-PSCs with solvent additives display relatively flat profiles. Note that for BHJ-PSCs without DIO, the vertical concentration profile is slightly unfavorable for regular architectures. Removing the use of solvent additive and generating the adequate vertical concentration gradient resulted in large increases in stability of the devices. Device stabilities are usually characterized under various conditions to extrapolate their lifetime. One of these tests is stability at high temperature. Here, the authors demonstrated that the DfBL-PSCs displayed only minor variations of PCEs up to 2 h at 130 °C. It is also safe to assume that much longer stable periods would be achieved if the authors left the devices for longer heating times. On the other hand, upon heating for 2 h, the BHJ-PSCs PCE decreased from 8.5% down to 3.5% (Figure 17b).

In summary, DfBL-PSCs have not yet been studied as extensively as BHJ-PSCs but still exhibit PCEs over 8.5%. Although these performances are lower than the best BHJ devices, the DfBL concept presents some major advantages with respect to the one-step deposition technique. For instance, doping of either the ED or the EA layer can be achieved independently to enhance the transport of a single carrier and lead to more balanced charge extraction in devices. On the other hand, while some ED–EA systems require the use of solvent additives, the highest performing DfBL-PSCs were fabricated without solvent additives or thermal annealing which considerably facilitates their production. Last but not least, due to their peculiar morphology and the fact that similar PCEs can be obtained without the use of solvent additives, DfBL-PSCs display much higher stabilities as compared to BHJ-PSCs. These results demonstrate that, although this has not yet been achieved, DfBL-PSCs have the potential to overcome the performances of BHJ-PSCs or, at least, provide an alternative deposition technique for ED–EA systems which do not perform as expected in BHJ-PSCs due to inadequate vertical concentration gradients.

## 4. Conclusions

In conclusion, we have reviewed a variety of processes that induce formation of adequate vertical ED–EA concentration gradients for PSCs in both single active layers and sequentially deposited multilayer active layers. Studies on the most commonly used ED–EA systems such as P3HT:PC_61_BM demonstrate that fullerene derivative-depleted layers are often found at the buried substrate/active layer interface. This vertical distribution adequate for iPSCs can be modified by using post-deposition processes such as thermal or solvent annealing. Depending on the substrate and top electrode material, a variety of distributions can be obtained which can be then used to fabricate higher performing regular and inverted PSCs. Processes such as surface treatment with solvents and solvent additives can also largely influence the formation of ED-rich or EA-rich layers at the active layer interfaces and FF as high as 67% can be obtained. Chemical modifications of ED or EA molecules is also a valid method to induce such gradients and strategies such as photo-polymerization of fullerene derivatives was proposed as an elegant method to produce adequate ED–EA distributions in either rPSCs or iPSCs depending on the side on which light is shone.

An alternative solution to produce concentration gradients is the sequential deposition of multilayers with varying ED–EA concentrations. This can be achieved in both regular and inverted devices either by dry processes such as transfer-printing or lamination, or by multilayer solution deposition. Dry processes ensure that no interdiffusion will occur between the top and bottom layers and, consequently, increase the control over the interface between the various layers. High efficiency devices were produced using this approach, which, however, involves additional fabrication steps. All solution approaches using either high Mw polymers or chemically modified fullerene derivatives also displayed positive results. More recently, a simple strategy based on diffusion of EA in the ED network was introduced and PCEs up to 8.6% in thermally stable devices have been achieved. DfBL-PSCs still have lower PCEs as compared to BHJ-PSCs. However, recent studies demonstrated that, as this alternative deposition process becomes more popular, increasing performances are obtained and DfBL-PSCs have the potential to overcome even the PCE of BHJ-PSCs.

## Figures and Tables

**Table 1:** Correlation between active layer thickness, poly(3-hexylthiophene) (P3HT) gradient and device parameters ^a^.

Active Layer Thickness	P3HT Gradient	Jsc (mA/cm^2^)	Voc (V)	FF (%)	PCE (%)
50 nm (unannealed)	N/A	2.6	0.55	60	0.8
50 nm (annealed)	Weak	3.4	0.58	58	1.2
100 nm (unannealed)	N/A	6.6	0.56	52	1.9
100 nm (annealed)	Strong	9.4	0.60	62	3.5
200 nm (unannealed)	No	5.5	0.53	56	1.6
200 nm (annealed)	No	5.6	0.57	61	1.9

^a^ Adapted from [30], with permission from © 2009 American Chemical Society.

**Table 2:** Effect of 1,8-diiodoctane (DIO) addition on the photovoltaic parameters of poly(2,6-Bis(trimethyltin)-4,8-bis(5-(2-ethylhexyl)thiophen-2-yl)benzo[1,2-b:4,5-b′]dithiophene): [6,6]-Phenyl-C_71_-Butyric Acid Methyl Ester (PBD-TTT-C-T:PC_71_BM) polymer solar cells ^a^.

Device Architecture	DIO (vol %)	Jsc (mA/cm^2^)	Voc (V)	FF (%)	PCE (%)
rPSC	0	13.1	0.84	49.8	5.48
rPSC	3	15.2	0.76	62.3	7.20
iPSC	0	14.2	0.81	45.1	5.19
iPSC	3	17.7	0.77	67.0	9.13

^a^ Adapted from [53], with permission from © 2014 WILEY-VCH.

**Table 3:** PV parameters of regular architecture polymer solar cells with alcohol/H_2_O soluble fullerene derivative top layers ^a^.

Fullerene Derivative	Doping	Jsc (mA/cm^2^)	Voc (V)	FF (%)	PCE (%)
-	-	9.02	0.60	67	3.61
EGMC-OH	-	9.43	0.60	66	3.71
EGMC-OH	CS_2_CO_3_ (40%)	9.71	0.60	65	3.74
EGMC-COOH	-	9.61	0.60	66	3.80
EGMC-COOH	Li_2_CO_3_ (40%)	10.9	0.60	66	4.29

^a^ Adapted from [85], with permission from © 2013 American Chemical Society.

**Table 4:** Effect of solvent additives on the performances of Poly[*N*-9′-heptadecanyl-2,7-carbazole-alt-5,5-(4′,7′-di-2-thienyl-2′,1′,3′-benzothiadiazole)]:[6,6]-Phenyl-C_71_-Butyric Acid Methyl Ester (PCDTBT:PC_71_BM) diffusive bilayer-polymer solar cells ^a^.

PCDTBT Layer	PC_71_BM Layer	Jsc (mA/cm^2^)	Voc (V)	FF (%)	PCE (%)
-	-	2.68	0.84	48	1.09
DIO	-	4.27	0.94	45	1.82
-	DIM	5.18	0.88	63	2.88
DIO	DIM	12.02	0.90	66	7.12

^a^ Adapted from [95], with permission from © 2015 Nature Publishing Group.
